# Bacterial Heterologous Expression System for Reconstitution of Chloroplast Inner Division Ring and Evaluation of Its Contributors

**DOI:** 10.3390/ijms19020544

**Published:** 2018-02-11

**Authors:** Hiroki Irieda, Daisuke Shiomi

**Affiliations:** 1 Academic Assembly, Institute of Agriculture, Shinshu University, Nagano 399-4598, Japan; 2 Department of Life Science, College of Science, Rikkyo University, Tokyo 171-8501, Japan

**Keywords:** chloroplast division, Z ring, membrane-tethering, heterologous expression, *E. coli*, AtFtsZ1, AtFtsZ2, ARC6, ARC3

## Abstract

Plant chloroplasts originate from the symbiotic relationship between ancient free-living cyanobacteria and ancestral eukaryotic cells. Since the discovery of the bacterial derivative *FtsZ* gene—which encodes a tubulin homolog responsible for the formation of the chloroplast inner division ring (Z ring)—in the *Arabidopsis* genome in 1995, many components of the chloroplast division machinery were successively identified. The knowledge of these components continues to expand; however, the mode of action of the chloroplast dividing system remains unknown (compared to bacterial cell division), owing to the complexities faced in in planta analyses. To date, yeast and bacterial heterologous expression systems have been developed for the reconstitution of Z ring-like structures formed by chloroplast FtsZ. In this review, we especially focus on recent progress of our bacterial system using the model bacterium *Escherichia coli* to dissect and understand the chloroplast division machinery—an evolutionary hybrid structure composed of both bacterial (inner) and host-derived (outer) components.

## 1. Introduction

Plant chloroplasts evolved from free-living cyanobacteria through primary endosymbiosis, which started with an engulfment of ancient cyanobacteria by ancestral eukaryotic cells approximately a billion years ago [1,2]. As in bacteria, the proliferation of chloroplasts is achieved by binary fission via a hybrid division machinery comprised of inner (stromal) bacterial and outer (cytosolic) host-derived elements [3,4,5]. This machinery mainly consists of four rings: two (the Z ring and the inner plastid-dividing (PD) ring) are inside, while the other two (the dynamin-related protein5B (DRP5B) ring and the outer PD ring) are outside the chloroplast [3,6,7]. Of those, the Z ring is broadly conserved from bacteria to chloroplasts [3,4,8,9,10,11]. Each Z ring is composed of FtsZ homolog tubulin-like GTPase, and is believed to generate a constrictive force for division both in bacteria and chloroplasts of *Arabidopsis thaliana* (although it has also been reported that the motive force was provided by the outer DRP5B ring, but not inner Z ring, in chloroplast of the red alga *Cyanidioschyzon merolae*) [8,9,10,12,13,14,15]. While the four rings and other division-related components might coordinately function to constrict the mid-chloroplast, the initial and critical event is the assembly of FtsZ into the Z ring just beneath the inner envelope membrane (IEM) at the future site of division. The Z ring then works as a scaffold, to which other components are recruited in a specific order to drive a division complex [4].

In contrast to the bacterial Z ring that is composed of a single FtsZ protein, the components of the chloroplast Z ring are two phylogenetically distinct FtsZ proteins, FtsZ1 and FtsZ2, which heteropolymerize into FtsZ filaments in vivo and in vitro [15,16,17,18,19]. FtsZ1 probably emerged from FtsZ2 through a gene duplication event, because the C-terminal amino acid sequence (which is critical for the membrane-tethering in bacterial FtsZ—see below) is conserved only in FtsZ2 [20,21]. FtsZ1 and FtsZ2 show high amino acid sequence identity and similarity regarding their GTPase core domain with bacterial FtsZ, but play distinct roles in the formation of the FtsZ polymers; FtsZ2 dominantly forms the backbone of the filament, while FtsZ1 assists in its remodeling [16,19,22]. In *A. thaliana*, FtsZ2 was additionally duplicated into two functionally redundant paralogs: AtFtsZ2-1 and AtFtsZ2-2. These two paralogs are functionally interchangeable with respect to in vivo chloroplast division activity, although the distinct contributions by these two AtFtsZ2 conforming to the shape of the chloroplast have also been reported [22,23].

The assembly and dynamics of the chloroplast Z ring is elaborately regulated by many components that negatively or positively affect the Z ring formation [3,4,5]. The stromal protein Accumulation and Replication of Chloroplasts 3 (ARC3) directly interacts with both AtFtsZ1 and AtFtsZ2 and inhibits the assembly of the Z ring at non-division sites [19,24,25,26], resembling the function of bacterial division inhibitor protein MinC for the positioning of the Z ring [27], whereas the IEM-spanning protein Accumulation and Replication of Chloroplasts 6 (ARC6) directly interacts only with AtFtsZ2 and promotes Z ring assembly in the stroma [22,26,28,29]. ARC3 and ARC6 were identified in the native AtFtsZ1-AtFtsZ2 complex isolated from *Arabidopsis* chloroplast [30]. Another IEM-spanning protein Paralog of ARC6 (PARC6) also directly interacts with AtFtsZ2, and in addition to the stromal proteins MinD and MinE, indirectly affects the Z ring formation through a direct interaction with the inhibitor protein ARC3 [25,26,31,32,33,34]. Briefly, in the working model of Z ring regulation in chloroplast division, ARC6 promotes the formation of a Z ring composed of AtFtsZ1-AtFtsZ2 heteropolymer, possibly by the tethering of AtFtsZ2 to the IEM. ARC3, MinD, and MinE act together as a Z ring positioning system and accurately confine the Z ring to the mid-chloroplast. During the remodeling and constriction of the Z ring, ARC3 may also function as an inhibitor of Z ring assembly after being recruited by PARC6 to the division site [3,4,5]. For an in-depth review of the many contributors to the chloroplast Z ring dynamics that include bacterial- and host-derivatives, we refer readers to previously published reviews [3,4,5].

To understand the chloroplast division comprehensively, in planta molecular analysis of Z ring assembly and dynamics is important. However, it can be challenging owing to the complexity of the plant cell, wherein many division-related components act together. Furthermore, plant breeding and genetic manipulation require more time compared to model microorganisms, even in the model plant *A. thaliana*. This situation has led to the development of some heterologous expression systems for *Arabidopsis* FtsZ proteins and other related components using single-celled model microorganisms, such as the yeasts *Schizosaccharomyces pombe* and *Pichia pastoris*, as well as the bacterium *Escherichia coli* [15,19,26,35]. The fission yeast *S. pombe* system was established as a cellular model for the functional analysis of bacterial actin-related protein MreB and FtsZ ahead of chloroplast FtsZ [36,37]. At present, together with recent methylotrophic yeast *P. pastoris* system, the yeast systems have shown the value of using heterologous expression systems for chloroplast division-related proteins—particularly filament and ring formation by FtsZs—to analyze their inherent functions [15,19,26,35]. On the other hand, based on the evolutionary background of the chloroplast and the fact that the Z ring-driven division system indeed functions in bacteria, as well as other practical advantages of a model bacterium, the *E. coli* system could be a good tool for the research of chloroplast FtsZ. However, the previous report showed that the chloroplast FtsZ produced in *E. coli* cells did not successfully form the Z ring or Z ring-like structure, but only formed long filaments and aberrant clusters; therefore, this system is lagging behind yeast expression systems [15,19,26,35].

**Figure 1:**
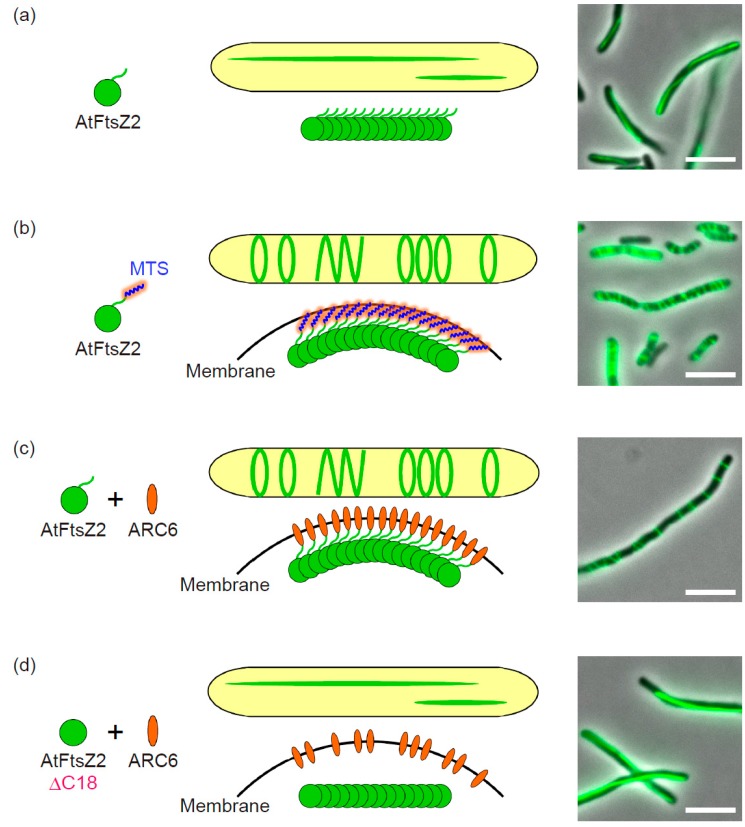
The formation of Z ring-like structures of *Arabidopsis* chloroplast FtsZ2 in the bacterial heterologous expression system. Schematic illustration of the proposed molecular behavior of chloroplast division-related components in *E. coli* cells and its merged microscopic image of phase-contrast and GFP are shown when expressing (**a**) super folder GFP (sfGFP)-AtFtsZ2, (**b**) sfGFP-AtFtsZ2-2MTS, (**c**) sfGFP-AtFtsZ2, and Accumulation and Replication of Chloroplasts 6 (ARC6) and (**d**) sfGFP-AtFtsZ2∆C18 (C-terminal 18-residue truncated form of AtFtsZ2) and ARC6. *E. coli* cells were grown in L broth (1% bactotryptone, 0.5% yeast extract, 0.5% NaCl) to the stationary phase at 22 °C. Scale bars: 5 µm. MTS: membrane-targeting sequence. To reduce the complexity in the diagrams, bundling of the FtsZ2 filaments was omitted.

Recently, we progressively developed the *E. coli* system to reconstitute Z ring or Z ring-like structures composed of the *A. thaliana* FtsZ protein AtFtsZ2-1 (hereafter called AtFtsZ2) [38]. Our system plausibly reflects the dynamic properties of AtFtsZ2, where the AtFtsZ2 assembles into long filaments or Z ring-like structures depending on the conditions. In Figure 1, we summarize the proposed molecular mechanism of action of AtFtsZ2 and its positive contributor ARC6, which has been demonstrated to anchor the chloroplast Z ring to the membrane in our system. In the following sections, we describe the development of this system and the important factors contributing to the filament morphology of AtFtsZ2, which includes N-terminal extended region of AtFtsZ2, membrane-tethering of the AtFtsZ2 filament, the negative regulator ARC3, and the positive regulators ARC6 and AtFtsZ1.

## 2. Optimization of *E. coli* System for Heterologous Expression of Chloroplast FtsZ

### 2.1. Fluorescent Tagging of AtFtsZ2 and Culture Condition

FtsZ proteins can polymerize, assemble into filament bundles, and eventually form the Z ring in the division system [3,4,5,8,9,39]. In each organism (bacterium or plant), visualizing the FtsZ protein using immunoelectron microscopy, immunofluorescence, or fluorescent protein (FP) labeling techniques clearly showed the Z ring formation at the division site [40,41,42,43,44,45,46,47]. In particular, FP labeling enables us to monitor the FtsZ protein dynamics in live cells. As for heterologous expression systems for chloroplast FtsZs tagged with FPs, in *S. pombe* yeast cells, AtFtsZ1 and/or AtFtsZ2 formed linear and ring-shaped filaments that were free-floating in the cytosol [19,35]. Furthermore, AtFtsZ1 and/or forcibly membrane-targeted AtFtsZ2 expressed in *P. pastoris* yeast cells assembled into ring structures, and the rings including membrane-tethered AtFtsZ2 showed contractible ability [15]. Thus, yeast systems can be available for the analysis of chloroplast FtsZs.

By contrast, as mentioned above, heterologous expression of AtFtsZ2 in *E. coli* cells only showed long filaments with aberrant clusters at 42 °C [19]. In bacterial cells, recombinant proteins frequently aggregate into inclusion-bodies because of high growth temperature and/or high-level expression of the heterologous protein [48]. In this context, we confirmed that the expression level of AtFtsZ2 protein introduced in *E. coli* was not too high, and successfully removed aberrant aggregation of AtFtsZ2 by decreasing the growth temperature to 22 °C—an optimal temperature for *A. thaliana* (Figure 1a) [38]. We also found that the growth phase of the bacteria strongly affected the AtFtsZ2 filamentation; sampling in the stationary phase is more suitable to form long filaments than in the logarithmic phase [38]. Some theories for this phenomenon include (i) reduced dynamics and a lower turnover rate of AtFtsZ2 compared with those of *E. coli* FtsZ (EcFtsZ) [19], and (ii) less competition with actively assembling EcFtsZ during the stationary phase. In the paper, we critically investigated the fusion terminus of FP [38]. In most papers to-date, irrespective of the derivatives from bacteria and plants, FPs were tagged at the C-terminus of FtsZ. On the other hand, as previously reported, we confirmed that N-terminal FP fusions of EcFtsZ were also precisely localized at the middle of the *E. coli* cell [38,49,50]. As for chloroplast FtsZ, we conducted heterologous expression of both N- and C-terminal FP-fused AtFtsZ2 and concluded that—at least in our *E. coli* system—filamentation of C-terminally FP-tagged AtFtsZ2 was repressed compared with that of an N-terminally fused one (Figure 1a) [38]. Since the C-terminal domain of FtsZ is important for its function, C-terminal FP-tagging might partially interfere with the filamentation ability of AtFtsZ2 [19,51]. Furthermore, the C-terminal FP fusions of AtFtsZ2 showed aberrant aggregations at higher temperature (37 °C) compared to N-terminal FP fusions [38]. As a consequence, we selected N-terminal FP-fused AtFtsZ2 for our expression system. Importantly, C-terminal FP fusions of FtsZ are generally assembly-competent, and the AtFtsZ2-FP fusion forms a Z ring at the middle of the chloroplast in *A. thaliana* [19,52]. Thus, this issue might need to be discussed. Many chloroplast proteins—including FtsZs—are encoded in the nuclear genome and are eventually transported into the chloroplast via its N-terminal transit peptide [12,45,46,53]. The transit peptide is cleaved upon its import into the chloroplast, and this is one reason why the C-terminal FP-fusion of AtFtsZ was preferred for in planta analysis [12,47]. Therefore, if the N-terminal FP-fused FtsZ is expressed in planta, it is necessary that the FP does not disturb the function of the transit peptide. In heterologous expression systems, the transit peptide-lacking chloroplast FtsZs are generally used, and this issue does not need to be considered.

### 2.2. N-Terminal Region of AtFtsZ2

Besides transit peptide, the chloroplast AtFtsZ2 protein harbors an extended N-terminal region compared with eubacterial FtsZ proteins [35,38]. AtFtsZ1 lacks this “extended” region, but many cyanobacterial and chloroplast FtsZ proteins exhibit N-terminal extension regardless of their amino acid sequence conservation, possibly implying the additional trait(s) in these FtsZs [38]. In the *S. pombe* yeast system, it has been reported that the N-terminal-extended region of AtFtsZ2 promotes its polymer bundling and turnover, suggesting that the N-terminus of AtFtsZ2—as with its C-terminus—is also important for its function [35]. Consistent with this, we confirmed the dependency of AtFtsZ2 filamentation on its N-terminus in *E. coli* cells, where the N-terminally-truncated AtFtsZ2 (here we deleted amino acid residues from 49 to 112 in addition to the transit peptide, AtFtsZ2∆N) with N-terminal FP showed considerably shorter filaments than did full-length FP-AtFtsZ2 fusion [38]. This suggests that the behavior of AtFtsZ2 produced in *E. coli* cytosol reflects its inherent properties.

### 2.3. Membrane-Tethering of AtFtsZ2

To establish the *E. coli* reconstitution system completely for the analysis of chloroplast division-related components, the formation of the Z ring composed of AtFtsZ2 in *E. coli* cells is critical. The FtsZ protein itself has no membrane spanning or anchoring domains and requires the cognate membrane associating and/or transmembrane proteins FtsA and ZipA in *E. coli* and Ftn2/ZipN in cyanobacteria, which interact with the C-terminus of FtsZ and target it to the membrane [42,54,55,56,57,58,59,60,61,62]. In green lineage chloroplasts, IEM protein ARC6—an Ftn2/ZipN ortholog—was believed to tether the Z ring, though no direct evidence has been reported for this so far [22,28,29,63,64]. *E. coli* has no ARC6 homolog, and FtsA and ZipA might not target AtFtsZ2 to the membrane, despite partial conservation of the FtsA-interacting sequence in the AtFtsZ2 C-terminus, which was supported by the fact that AtFtsZ2 did not form ring-like structures (Figure 1a) [38].

Previous reconstitution systems using liposomes revealed that the membrane-tethering of FtsZ is required for Z ring formation, in which reconstitutions of contractile EcFtsZ ring were achieved by artificial membrane-tethering with the C-terminal membrane-targeting sequence (MTS) or natural tethering through a co-introduced FtsA. This membrane anchored EcFtsZ could actually constrict a liposome, indicating that membrane-tethering is critical to form the *E. coli* Z ring and generate a constriction force [14,62]. Similarly, in our *E. coli* system, an artificial membrane-tethering of AtFtsZ2 by MTS gave AtFtsZ2 the ability to form multiple Z ring-like structures in both wild-type and *ftsZ*-depleted *E. coli*, indicating the intrinsic property of AtFtsZ2 to form Z ring-like structures in *E. coli* cells (Figure 1a,b). However, these Z ring-like structures did not constrict a cell (probably because AtFtsZ2 could not interact with FtsA and ZipA, which stabilize Z ring composed of EcFtsZ) [38]. Around the same time, it was independently shown that the MTS-tagged chloroplast FtsZ derived from *Galdieria sulphuraria* thermophilic red alga (GsFtsZ) formed multiple Z ring-like structures in *E. coli* cells [65]. Together with the recent success in the *P. pastoris* yeast system that showed reconstitution of the ring-like structure of AtFtsZ2 by MTS-tagging [15], these reports strongly demonstrated that membrane-tethering is a necessary and sufficient factor for bacterial and chloroplast FtsZ proteins to form Z ring or Z ring-like structures.

Interestingly, the diameter of the ring-like structure formed by membrane-tethered AtFstZ2 was much larger than that of EcFtsZ when reconstituted in *P. pastoris* cells, resembling the size of their corresponding rings in vivo [15]. This has suggested that the structure of each FtsZ protein determines the curvature, and consequently the size, of each ring [15]. However, we successfully reconstituted the Z ring-like structures of AtFtsZ2 in *E. coli* cells [38]. Furthermore, in *A. thaliana*, the chloroplast size increases during leaf development, and leaf epidermal cells contained small chloroplasts with smaller AtFtsZ1 and AtFtsZ2 rings compared to leaf mesophyll cells [52,66]. Thus, we presume that FtsZ proteins have the potential to form Z rings with various diameters according to the cell or chloroplast diameter.

## 3. The Function of Negative and Positive Contributors in Bacterial Reconstitution Systems

### 3.1. The Negative Regulator ARC3

**Figure 2:**
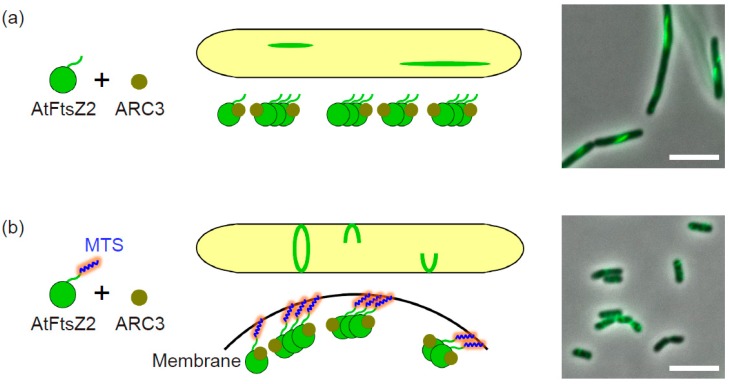
The effects of Accumulation and Replication of Chloroplasts 3 (ARC3) on the filaments of *Arabidopsis* chloroplast FtsZ2 in the bacterial heterologous expression system. Schematic illustration of the proposed molecular behavior of chloroplast division-related components in *E. coli* cells and its merged microscopic image of phase-contrast and GFP are shown when expressing (**a**) sfGFP-AtFtsZ2 and ARC3, and (**b**) sfGFP-AtFtsZ2-2MTS and ARC3. *E. coli* cells were grown in L broth (1% bactotryptone, 0.5% yeast extract, 0.5% NaCl) to the stationary phase at 22 °C. Scale bars: 5 µm. MTS: membrane-targeting sequence. To reduce the complexity in the diagrams, bundling of the FtsZ2 filaments was omitted.

In rod-shaped bacteria such as *E. coli* and *Bacillus subtilis*, mid-cell positioning of the Z ring is tightly regulated by the Min system, in which the negative regulator MinC inhibits FtsZ polymerization at cell poles [67,68]. Spatial regulation of the Z ring by MinC is also conserved in cyanobacteria [69]. In contrast, except for certain algal lineages and the moss *Physcomitrella patens*, the chloroplast has no MinC homolog, but instead acquired the plant-specific stromal protein ARC3 as a functional analog of bacterial MinC [19,24,25,26,52,70,71]. Indeed, *Arabidopsis arc3* mutants showed multiple Z rings and nonuniform chloroplast size and number, whereas ARC3-overexpressing mutants exhibited a small number of enlarged chloroplasts with fragmented AtFtsZ filaments [25,26,72,73]. ARC3 directly interacts with both AtFtsZ1 and AtFtsZ2, and these interactions were inhibited by the C-terminal membrane-occupation-and-recognition nexus (MORN) domain of ARC3 [25,26]. The MORN domain is a binding site of PARC6, which is believed to recruit and activate ARC3 at the chloroplast division site [32,34]. In a yeast heterologous expression system which does not contain the PARC6 homolog, recombinant ARC3 lacking the MORN domain was used to analyze the ARC3 inhibitory effects on AtFtsZ filaments [19,26]. We also co-expressed this mutant ARC3 and AtFtsZ2 with an FP in *E. coli* cells and evaluated its function in our bacterial system. Consistent with previous reports, we confirmed the inhibition of the AtFtsZ2 assembly by ARC3 regardless of the presence or absence of the MTS tag (Figure 2a,b) [38]. Since it has already been reported in the yeast system that ARC3 inhibited the assembly of cytosolic free-floating AtFtsZ filaments (linear and ring-shaped structures), our bacterial system presented the first example of ARC3 in inhibiting AtFtsZ filaments in membrane-tethered Z ring-like structures in a heterologous expression system (Figure 2b) [19,26,38]. It is worth noting that in our *E. coli* system— like the yeast systems—there might be no factors that affect ARC3 behavior. The consistency of the inhibitory effects of ARC3 on FtsZ filament assembly among yeast, *E. coli*, and in planta analyses strongly demonstrates the clear function of ARC3 in chloroplast Z ring regulation. This is further supported by a recent study in which in vitro assays showed that ARC3 promoted AtFtsZ2 debundling and disassembly by enhancing its GTPase activity and 3D reconstruction using single-particle analysis, suggesting that PARC6 mediated ARC3–AtFtsZ2 interaction [74]. Bacterial MinC also promotes the debundling and disassembly of FtsZ, but does not affect its GTPase activity [27]. In addition, chloroplast ARC3 binds to both MinD and MinE, but bacterial MinC only binds to MinD [25,75]. The analogous function of ARC3 to MinC is indisputable, but there might be differences between ARC3 and MinC in their mode of action in each division system.

### 3.2. The Positive Regulator ARC6

Using liposomes and purified EcFtsZ proteins, Osawa and Erickson (2013) demonstrated that the reconstitution of the Z ring (or Z ring-like structure) membrane-tethered by its natural partner is one goal for protein-free or heterologous expression systems in order to study the molecular mechanisms of the Z ring-centered division machinery [62]. In the case of chloroplast division machinery, a Z ring-anchoring factor has not yet been identified, but a great deal of indirect evidence implied the IEM protein ARC6 as a potential candidate [22,28,29,34,45,63,64]. In *Arabidopsis*, FP-labeled ARC6 concentrated at the chloroplast constriction sites in the shape of a ring, and ARC6 mutants exhibited a Z ring-defective phenotype, consequently leading to a small number of enlarged chloroplasts [28,45,63,64]. Using the yeast two-hybrid system, a direct interaction between ARC6 and the C-terminal conserved sequence of AtFtsZ2 was demonstrated [22,29,34]. FP-labeled ARC6 and AtFtsZ2 co-localized in the yeast cytosol, which mostly depended on the AtFtsZ2 C-terminus [34]. Collectively, all these reports revealed that ARC6 is a positive regulator of Z ring formation in chloroplasts. Therefore, the next challenge will test whether ARC6 truly anchors the chloroplast Z ring to the membrane.

We applied Osawa and Erickson’s strategy to our *E. coli* heterologous expression system, where the MTS-untagged AtFtsZ2 (which itself can only form linear filaments) and ARC6 were co-expressed, and evaluated the effects of ARC6 on AtFtsZ2 filament morphology [38]. Fortunately, our challenge was a success—AtFtsZ2 polymer drastically altered its morphology from the linear filaments to Z ring-like or helical structures dependent on ARC6 (Figure 1a,c). FP-labeling of both AtFtsZ2 and ARC6 showed co-localization of these two proteins in the ring-like structures [38]. These Z ring-like structures completely depended on the ARC6-interacting sequence at the AtFtsZ2 C-terminus (here we truncated 18 amino acids, AtFtsZ2∆C18), suggesting ARC6-mediated tethering of AtFtsZ2 filaments to the membrane (Figure 1c,d). Membrane-fractionation assays further supported the membrane attachment of AtFtsZ2 by ARC6 through their direct interaction [38]. The C-terminal region of ARC6 protrudes into the chloroplast IEM and directly interacts with the outer envelope membrane (OEM) protein Plastid Division 2 (PDV2), being able to transfer the Z ring positioning information from the stromal division machinery to the cytosolic one [76,77]. Together with previous results, our data obtained from the bacterial reconstitution system clarified that the other N-terminal side of ARC6 interacts with AtFtsZ2—a backbone protein of the Z ring through which ARC6 directly anchors the Z ring to IEM [22,28,29,34,38,45,63,64]. Bacteria such as *E. coli* and *B. subtilis* have no ARC6 ortholog, but the membrane-interacting protein FtsA interacts with FtsZ and anchors it to the membrane, hence stabilizing the Z ring [42,57,58,62,78,79]. By contrast, cyanobacteria uniquely evolved Ftn2/ZipN—an ancestor of chloroplast ARC6—as a functional analog of FtsA for Z ring-tethering [59,60,61]. The successful reconstitution of chloroplast FtsZ2 ring in bacterial cells by membrane-tethering through the chloroplast ARC6 indicates high stability and plasticity of the Z ring-centered division machinery that is conserved from bacteria to chloroplasts.

Additionally, in the *S. pombe* yeast expression system, it has been demonstrated that ARC6 stabilizes AtFtsZ2 filaments independent of its tethering ability [35]. On the other hand, a recent report revealed a new function of the *E. coli* FtsA in aligning FtsZ protofilaments in the unbundled state and stabilizing them, in addition to its membrane-tethering ability [80]. Thus, these functional analogs commonly work for Z ring-tethering but have additional function(s) as a positive regulator in each division system.

### 3.3. The Positive Regulator AtFtsZ1

**Figure 3:**
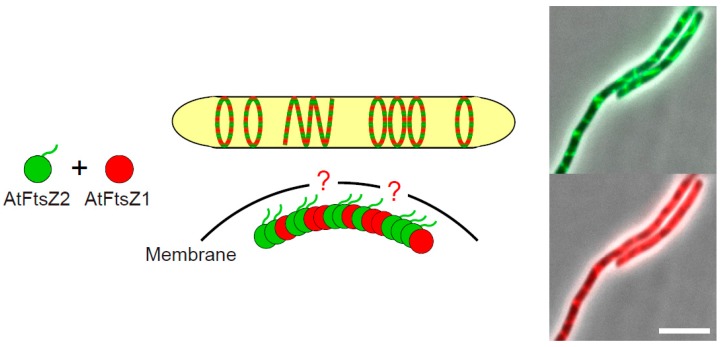
The effects of *Arabidopsis* chloroplast FtsZ1 on the formation of Z ring-like structures of *Arabidopsis* chloroplast FtsZ2 in the bacterial heterologous expression system. Schematic illustration of the Z ring-like structures composed of AtFtsZ1 and AtFtsZ2 heterooligomer in *E. coli* cells, although the mechanism by which the *Arabidopsis* FtsZ filaments are tethered to the membrane is unclear (indicated by red question marks in the illustration), and its merged microscopic images of phase-contrast and GFP (upper panel), and phase-contrast and mCherry (lower panel) are shown. *E. coli* cells expressing sfGFP-AtFtsZ2 and mCherry-AtFtsZ1 were grown in L broth (1% bactotryptone, 0.5% yeast extract, 0.5% NaCl) to the stationary phase at 22 °C. Scale bar: 5 µm. To reduce the complexity in the diagram, bundling of the FtsZ filaments was omitted.

In our paper, we described the unexpected function of AtFtsZ1 in positively contributing to the filament morphology of AtFtsZ2 in the bacterial expression system [38]. As mentioned above, the chloroplast Z ring is composed of AtFtsZ2 and AtFtsZ1, but the former dominantly determines the filament morphology, while the latter plays a regulatory role [15,19]. It is worth noting that the *Arabidopsis ftsZ1* mutant (like *ftsZ2* mutants) showed a small number of enlarged chloroplasts, implying the indispensable function of AtFtsZ1 in chloroplast division, although it has also been observed that chloroplasts in the *ftsZ1* mutant still exhibited a single mid-plastid constriction [22,26,30,81]. As expected, FP-labeled AtFtsZ1 and AtFtsZ2 co-localized in the *E. coli* expression system, consistent with previous observations that AtFtsZ1 and AtFtsZ2 can form a heteropolymer both in vitro and in yeast systems (Figure 3) [15,17,18,19,38]. However, the AtFtsZ1 surprisingly induced a morphological transformation of AtFtsZ2 filaments into ring-like and helical structures in *E. coli* cells, resembling Z ring-like structures tethered by ARC6 (Figure 3) [38]. This phenomenon was also observed in the case of AtFtsZ2∆C18 [38]. AtFtsZ1 possesses no C-terminal sequences responsible for membrane-tethering, and independently expressed AtFtsZ1 showed only a dispersed pattern in the bacterial cytoplasm, which suggests that AtFtsZ1 itself is unlikely to interact with any of the *E. coli* endogenous components supporting membrane-tethering [38]. Thus, it still remains an open question as to how AtFtsZ2 filaments form Z ring-like structures depending on AtFtsZ1 in the bacterial expression system. In the *P. pastoris* yeast system, a ring-like structure of MTS-untagged AtFtsZ1 was observed in the absence of other related component(s), although we did not detect any AtFtsZ1 filaments or rings in *E. coli* cells [15,38]. As for membrane-tethering, these data led to the speculation that AtFtsZ1 itself may interact with the membrane. Nevertheless, it is clear that AtFtsZ1 works positively to form the chloroplast Z ring. The conservation of two distinct FtsZ proteins in green lineage indicates a unique mechanism to regulate chloroplast Z ring assembly and dynamics compared to bacterial cell division. We hope future studies will reveal unknown mechanisms of action of AtFtsZ1 apart from its ability to increase the FtsZ filament turnover rate [19].

## 4. Conclusions

The *E. coli* reconstitution system has now become one of the many useful heterologous expression systems for studying the FtsZ-centered chloroplast division machinery. Although in planta analysis is the best way to comprehensively examine the components employed in chloroplast division, heterologous expression systems lacking such native division-related contributors also provide important insights into inherent properties of the introduced component(s). In general, microbial manipulations have practical advantages, such as rapid growth, axenic culture, and genetic accessibility. Yeast systems allow the advantage of a eukaryotic environment completely lacking plant derivatives. The advantages of bacterial systems are as follows: (i) bacteria is an evolutionary progenitor of chloroplast and bacterial cytosol, wherein the Z ring-centered division system still works and is topologically equivalent to chloroplast stroma; and (ii) bacteria—especially *E. coli*—are distantly related to cyanobacteria, and partly lack the homologs of chloroplast division-related proteins. Indeed, using the *E. coli* system, we reconfirmed the negative regulation of the chloroplast FtsZ2 filaments by plant-specific ARC3 and found that this regulation was also effective for the membrane-tethered one (Z ring-like structures) [38]. We also directly demonstrated the Z ring-tethering ability of ARC6, which is unique to cyanobacteria and chloroplasts [38]. The components of the chloroplast division machinery in the stromal side (including IEM) have changed over the course of evolution [3,4,5]. Besides ARC3 and ARC6, the homologous proteins of PARC6 and MULTIPLE CHLOROPLAST DIVISION SITE 1 (MCD1) are absent in *E. coli* [32,33,34,82]; hence, they are capable of being applied to our bacterial system. Additionally, bacteria-derived chloroplast MinD and MinE can be analyzed by using the minCDE deletion mutant of *E. coli*, which creates minicells but also shows normal- and long-sized cells, as in the case of AtFtsZ2 expressed in the ftsZ-depleted *E. coli* strain [38,83]. By contrast, in the bacterial system (and in the yeast systems), the analyses of division-related components employed in the plant cytosolic side (including OEM), such as DRP5B, Plastid Division 1 (PDV1), and PDV2, appear more challenging [7,13,76,77,84,85]. Accordingly, the integration of data obtained from yeast and *E. coli* systems into in planta results is important, and will continue to accelerate the research on chloroplast division.
